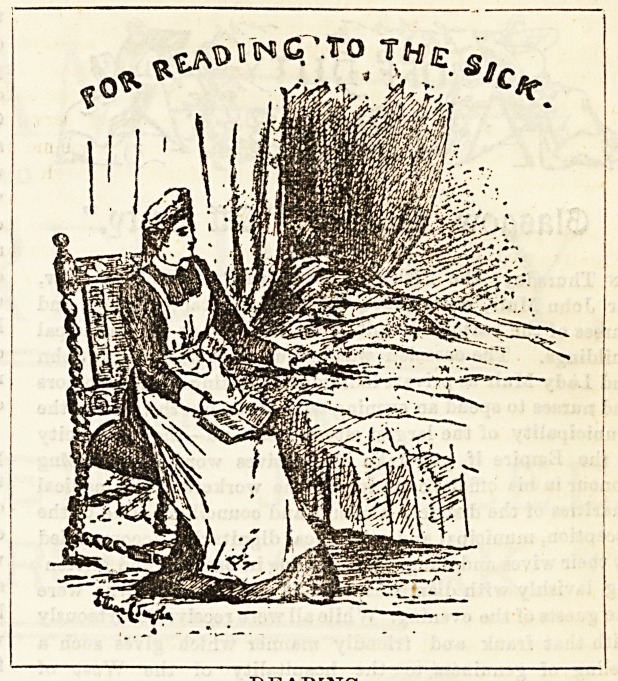# The Hospital Nursing Supplement

**Published:** 1892-11-05

**Authors:** 


### "The Hospital" Nursing Mirror.

Being the Extra Nursing Supplement of "The Hospital" Newspaper.

[Contributions for this Supplement should be addressed to the Editor, The Hospital, 140, Strand, London, W.C., and should have the word "Nursing" plainly written in left-hand top corner of the envelope.]

### En Passant.

HULL DISTRICT NURSES.—We are very glad to hear that an influential committee is doing its best to gain subscribers to a district nurses' home in this great town. Hitherto the only nursing among the poor has been carried on at the expense of one lady, but it is proposed to get enough money together to enable the committee to start working with at least five nurses.

LEEDS DISTRICT NURSES.—The *Leeds Mercury* contained last week an excellent account of the work done by this association. The writer traced the origin and growth of the work from twelve years ago, when three nurses only were employed, who lived in a little house in Caledonian Road, to the present day, when eight nurses are working under Miss Brown, and many more are needed before the whole of Leeds can be embraced in the scheme. Three nurses, we are told, will soon be working at Holbeck, where, thanks to Mrs. Meynell-Ingram, a home is about to be built, and where local enthusiasm has raised £2,000 to support it. A good account of a district nurse's day closed a good article, which will, we hope, result in the gain of many kind friends for the work.

STOCKTON AND THORNABY.—Fourteen months ago the Nursing Association of this neighbourhood was started, and it has gone on and succeeded in a wonderful manner, and has five nurses, including the superintendent nurse, on its books at the present time. One nurse has been set apart to pay nursing visits at a shilling a visit, or five shillings a week, so that those who cannot afford the fees charged by a permanent nurse, but can afford a small payment, are able to avail themselves of the services of a trained nurse who can stay long enough to do all that is necessary to supplement the skill of the doctor. There is one especial point in connection with this association which is very gratifying, and which says more in praise of the suffering relieved by the nurses than anything else could. The men at twenty-one works in the neighbourhood, numbering 8,000, are systematic contributors to the maintenance of the present work of the institution.

DUNDEE DISTRICT NURSES.—Many accounts of the Dundee festivities on the occasion of the visit of Princess Louise to the city have reached us, and we are glad to hear of the pleasure the nurses at the Dudhope Street Home enjoyed through the visit. The seven nurses now working at the home were with Miss Mackay, the Lady Superintendent, presented to the Princess, who spoke to them very kindly of the good work they are doing. The annual meeting of the Dundee Sick Poor Nursing Association was held on October 24th, and was very largely attended. The work is spreading rapidly, and in the two and a-half years in which the society has existed the number of nurses has been increased from two to seven, and now the time has come when the accommodation for the nurses must be increased and when funds are required. The nurses paid 21,316 visits in the year, relieving pain and weariness in many homes. We do hope earnestly that some large donations will be forthcoming to help increase this society, which is affiliated to the Q.V.J.I.N., and that the visit of the President of the Scottish branch will bring its good work to the notice of many who have never yet helped it on by so much as a shilling.

THE GUILDFORD INSTITUTE.—Lady Alice des Voeux and a few of her friends have started a Nurses' Institute and Home for Private Patients in Nightingale Road, Guildford. Miss Truscott has been appointed matron, and four nurses are under her, and their number will be increased according to the demand. The home stands on the top of the hill, and there is a fine view from the windows. Patients of both sexes will be admitted, and may be attended by whatever medical man they may choose to select.

THE SARAH ACLAND HOME, OXFORD.—Mrs. W. E. Gladstone paid a visit to this home on Tuesday, the 25th of October. On her arrival she was given a beautiful bouquet of red roses by the Hon. Mrs. E. Talbot. After being shown through the patients' rooms by Miss Denniston (Lady Superintendent) she visited the nurses in their sitting room. They were very much interested in the account she gave them of her experience of cholera whilst visiting the wards of a London hospital during the epidemic. Sir Henry Acland, the Hon. Mrs. E. Talbot, Mrs. Turner (Vice-President), and Miss Bull (Secretary) were present.

INCURABLE CHILDREN.—We are glad to hear of another home opening its door to receive these helpless little mortals, whose life is very hard at best, and for whom there seems so terribly little accommodation. The Sisters of St. Mary's Lodge, Halton, in Hastings, have opened a ward in their home, where they will receive girls from the age of five to ten years of age. Chronic or incurable cases of any sort, such as hip disease or paralysis, will be admissible, and they will be nursed by the Sisters. The payments will be from 5s. 6d. to 7s. 6d. a week, according to the treatment required. The ward has been opened principally for the relief of children of the middle-class. Sister Cordelia will be very glad to answer any questions concerning the children, or give any required information.

LOWESTOFT PROVIDENT NURSES' FUND.—The fourth annual meeting of the committee and subscribers of this association was presided over by Sir Philip Cunliffe Owen. We are glad to learn that the committee have been sending out one or two of their nurses as district nurses, as a tentative measure merely, but it has met with so much success, that we hope it will speedily develop. There is a most respectable balance in hand, but the committee are wisely keeping that for the rainy day which invariably comes when we have no provision for it. Lady Cunliffe Owen, the President of Committee, is desirous of starting a fund with which to provide dinners and invalid food for those who cannot possibly afford to buy any sort of nourishing food. Such a scheme requires the utmost care and supervision, but wisely administered could prove a great good.

TESTIMONIALS AND PRESENTS.—Miss Tudor writes a sensible article on this vexed question in *Nursing Notes*, and points out very justly that presentations are fast becoming a nuisance, and more than that to the nurses, whose earnings have often to go to providing necessities for those at home. Where certain festivals, such as birthdays, have become well-known and time-honoured institutions, Miss Tudor suggests that the subscription to the gift shall be limited to sixpence. Where there are great numbers of nurses, we think it might be limited to half that sum, not because we wish nurses to appear generous and really be at small cost to themselves, but because we know to a certainty that many of our brightest and most kindly nurses, who always appear ready to join a subscription at any time, really need every penny of their quarter's money for pressing private needs.

### Lectures for Asylum Attendants.

By William Harding, M.B.

### VI.—SOME IMPORTANT POINTS; EXCRETIONS, &c.

The nurse into whose ward a patient is placed on admission will as early as possible obtain a sample of that patient's urine in order that it may be examined. This should be done in every case as a matter of ordinary routine. It is not always easy to get a specimen, owing to the patient's mental condition. An effort, however, should always be made to get enough for an examination, and at times it is necessary to collect the total amount passed in the twenty-four hours for measurement; this will require the patient to be kept under the closest observation. The nurse who has charge of the dormitory should notice each morning and observe whether there be anything unusual in the urine of the respective patients as regards quantity, colour, or sediment. If there be anything out of the common she will save the specimen and report the occurrence to the charge nurse. Frequency of micturition or any difficulty or pain in doing so should be noted. In the male wards this is more especially important, since the patient may be suffering from stricture of the urethra which prevents him passing urine. An excitable or violent patient with a distended bladder is in a dangerous condition, and in the event of a struggle serious injury may unintentionally be done him. The bladder when distended is easily ruptured, and this accident might readily occur in the case of a patient attacking an attendant or other patient. More especially might it be likely to happen should the struggling pair fall to the ground. The charge attendant should on admission, or as early as possible afterwards, note the manner in which a newly-arrived patient passes urine, whether it is passed with ease or with much straining and difficulty.

It is often almost impossible, even in presumably sane individuals, to get accurate information as to the state of the bowels. Many women who ought to know better are dreadfully careless of themselves in this respect, and constipation is a very common affection among the female lunatics. It is frequently associated with anæmia, and, indeed, helps to keep up that condition. In all cases, but especially amongst epileptics, the nurse should take notice whether any of her patients suffer in this way. We every day meet lunatics who declare that they are purged while passing normal motions. There are others who, with bowels freely moved, affirm that they are obstinately constipated, and demand a daily purgative. Observation alone can decide as to the truth of these statements, and they should always be investigated. The patient, though often making false complaints, may on that particular occasion be really suffering. The first symptoms of colic or any diarrhœa should be at once reported. This is especially important, as abdominal affections in the insane are often very obscure. The character of the diarrhœa should be noticed, with particular regard as to whether there be any blood in the motion. The nurse should also notice whether the patient has anything in the nature of piles, for even should the doctor think it necessary to make an examination, with some patients it is very convenient to have a preliminary examination made by the nurse. No patient with purging or abdominal pain should be given the ordinary diet until the case has been reported and instructions as to diet, &c., received. Whenever possible, the patient's temperature should be taken, and form part of the report. This is a matter of the utmost importance, seeing that the treatment of the form of dysenteric diarrhœa sometimes met with among the insane is successful in proportion to the earliness of the stage of the disease when the patient is put under treatment. Extreme cleanliness and early attention to the slightest abdominal symptoms will reduce these cases to a minimum.

The first appearance of menstruation after a patient's admission should be duly reported. In all cases anything unusual as regards the amount or frequency should be noted, and also whether it is accompanied by pain or is attended by any marked change in the patient's mental condition. The nurse should also be careful to report the presence of any abnormal discharges. Another point which must have the nurse's attention is whether there is any excess in the amount, or any peculiarity in the distribution of the sweat, as, for instance, whether it be confined to one limb or to one side of the body, &c.

Vomiting is not an infrequent symptom among the insane. There are idiots who can with ease regurgitate their food, and, indeed, like ruminants, appear to chew the cud. Some patients eat too rapidly or too much, and vomit almost at will. There are others who, from hysterical ideas or from sheer stupidity, will induce vomiting by putting their fingers down their throats. The appearance of any nausea, paleness, or sweating before vomiting should be noticed; also whether the act was performed easily or with difficulty. The nurse should examine the matter vomited for blood, foreign bodies, &c. Sometimes buttons, pieces of straw, or such-like articles, may be found. If there be anything unusual it should be kept in order that the doctor may inspect it. Especially with regard to hernia is vomiting to be borne in mind. Attempts at vomiting are sometimes the only symptoms of a strangulated hernia in a dement. At bath, and on every occasion when the nurse has an opportunity of observing, she should take notice whether there is any swelling or fullness in the regions where hernias are commonly found. It is possible that she may find mare's nests, such as an enlarged inguinal gland, but such things have an importance of their own, and at the same time the nurse shows that she is taking an intelligent interest in her work. Any blood, whether brought up by coughing or vomiting, should be carefully saved with whatever came up with it. Patients will sometimes declare that they have coughed or vomited up blood when it only proceeds from the gums. In these cases the pocket-handkerchief or apron stained should always be kept and submitted to the doctor.

### Mary Wardell Convalescent Home for Scarlet Fever.

Eight years ago the Mary Wardell Convalescent Home for Scarlet Fever was formally opened by the Prince and Princess of Wales, who were accompanied by their three daughters. Since then a vast amount of valuable work has been done in the charming house which is situated on Brockley Hill, one of the loveliest spots existent in a neighbourhood justly celebrated for its fine views and healthy air. The house has extensive private grounds, which are, of course, an absolute necessity for patients recovering from a highly infectious disease.

The home has an omnibus of its own, which is always sent to convey persons from the hospital or house where they have passed through the acute stage of the disease. This omnibus naturally necessitates the maintenance of a man and a horse, and the garden needs a gardener, and the disinfecting apparatus (a very satisfactory piece of machinery by Benham) requires skilled management. A laundry, located in a separate building, is another branch to be worked with special care and intelligence, all infected linen being conveyed direct thither in covered pails, to emerge purified and clean by a different route, and thence, in distinct baskets, to be taken to the well-ordered linen-room for redistribution. All these and many other arrangements make the Home a costly one to work; and the payments made by patients do not, and never will, cover anything like the annual expenses. Of course, regular visits from the medical officer and the presence of a trained nurse are amongst the absolute necessities of such an establishment. Of the value of such a place no one has ever expressed a doubt, and the organisation of the scheme met with most cordial approval from many leading members of the medical profession, but amongst the general public the knowledge of and interest in the undertaking are terribly limited.

For some inexplicable reason, fever hospitals are always alluded to with bated breath, and since the charming ceremony of opening the Mary Wardell Convalescent Home took place only patients and their near relatives, medical men, and members of the committee have kept up much personal knowledge of the work. After being temporarily closed for necessary fumigation, white-washing, painting, and repairs, Miss Mary Wardell acquainted all interested in the undertaking, through the medium of the *Times*, &c., that on October 27th and 28th the Home would be opened for inspection, and a sale of work would take place prior to the readmission of patients. It was an exceptional opportunity for outsiders to penetrate the mystery, and see how things were managed in the only Home for convalescent scarlet fever cases in England, but, unhappily, both days were disastrously wet, which thinned the number of visitors considerably. The house itself leaves nothing to be desired, the rooms are light and airy and pretty, although naturally there are no unnecessary or germ-attracting adornments. Still, there is no ugly bareness noticeable. The colours of the walls are so pleasant, and the furniture is pretty, every article striking one as most suitable for the purpose it is designed for. Each window—and there are many—has a wondrous view of wooded country, and distant hills and sweet fresh air seem laden with strength and health for the invalids who come in search of both.

Seventeen hundred patients were admitted during the first eight years of the Home's existence, and a great many more have had to be refused from lack of space. With such a record of cases, it needs but little imagination to see how many home circles have benefited by the advantages it offers. The good done is so widespread, we fail to grasp its entirety at first; we think only of a household saved from an epidemic by the prompt removal of the first child who sickens, into a hospital first, and thence to this beautiful country house, where he is safely housed and cared for until he is strong and absolutely safe to mix again with his brothers and sisters. A contingent of school boys, too, often finds its way to Brockley Hill (which, by the way, is two miles from Stanmore, beyond Harrow, and only ten miles from Hyde Park), and a very jolly party they must make in the latter days of their convalescence. Well, the schools benefit, and so do the families, but we must not forget the safety also insured to the general community by this absolute, but not disagreeable, isolation of the scarlatina victims. We must remember that these benefits certainly confer a corresponding obligation on all of us. We are, in honour bound, to return to the best of our power, the good we thus receive—we owe, yes every one of us, a debt of gratitude to Miss Mary Wardell and those influential friends who are her steadfast supporters, for the seventeen hundred cases which she has helped back to health under circumstances which have insured the safety of a far larger number of persons, whom she has been instrumental in protecting from the dangers of infection. The scheme of the Home having been so well received and encouraged, it is a great surprise to us to find how insufficient has been the pecuniary aid subsequently forthcoming. Where are the thankofferings from those who have escaped scarlet fever, and where is the gratitude of those who have recovered from it? Miss Wardell has not only given largely of her own private means, but she has given herself to the work. Those who live amongst infectious cases have practically to renounce the pleasures of intercourse with the uninfected, for friends dread the very name of the disease, and make an alien of her who dwells amongst the sufferers. Although we are not all called upon to give our personal service, yet we can each one of us offer either sympathy or countenance, as well as subscriptions, to the enlargement and to the carrying-out of this most desirable work. It is a discredit to us that there should be only one such Home to receive the many thousands of annual patients for whom adequate and safe provision is unluckily needed. But it is still more of a discredit to find that the one Home already existent is in debt. Surely it is only want of thought, and not callous ingratitude, which leads so many, who have themselves benefited, coldly to ignore their obligation to render equal advantages possible for those who are too poor and friendless to secure them for themselves. Miss Mary Wardell's report is a most interesting and also practical one, and we hope our readers will study for themselves the details of the management. It is only possible to speak fairly of such an establishment after seeing and studying it, and we wish that a far larger number of visitors had taken advantage of the "open days," when the unusual opportunity of inspecting, without any personal risk, was offered to all. Particulars can be obtained by writing to the Hon. Secretary, Miss Mary Wardell, Convalescent Home, Stanmore, Middlesex.

### A Nurse's View of Christmas Decorations.

We all decorate our wards nowadays more or less just according to our taste, our own means, and the encouragement which we receive. If we keep our wards in good order all the year round, and take care of our growing shrubs and plants, we find that the addition of a moderate amount of holly and other evergreens secures a festive appearance at a moderate expenditure of time, labour, and money. Patients like the decorations, and they enjoy the preparation, and they are proud for their own friends to see the completed adornments of their wards. Thus we work away beforehand with goodwill and hearty support from our charges, and we all feel the better for the festival if we succeed in giving a pleasant aspect to it for those in whose monotonous lives "keeping Christmas" has been a meaningless phrase heretofore. But there is another side to the question, for is there not the probability that the elaborate devices which have to be commenced in November may become a weariness to nurses? At a time of year when there is so much serious sickness no unnecessary burdens should be laid upon workers lest the patients should suffer. Patients' comforts are the first consideration, and it is unnatural and unpardonable that the concoction of any device, however tasteful, should be allowed to absorb the attention which belongs of right to our sick people. Sometimes a ward sister has personal friends who are glad to employ their leisure hours in decorating her ward, and if they can undertake it entirely it is often a capital arrangement, and leaves the nurses free to continue their own duties. But however it is managed it ought to be done within the few days which precede Christmas; it is bad management to let the work hang about until the patients, to say nothing of the nurses, are tired before the day itself arrives.

### Our Indian Letter.

There is very little nursing news to send, but an extraordinary thing occurred to me last week which will show how natives mix up the relative duties of the female doctor and the trained nurse. One afternoon a native ward servant came and informed me that two Baboos wished to see me in my room. Visions of all sorts of things flashed through my brain as to what they could want. One was an old man with grey eyes and grey hair, not very dark. He was in native costume, with a bright orange turban or pugaree on his head. The other was young and dark; he was a native M.D., Dr. Ram. They salaamed, I bowed. The younger spoke good English, and approached with his hand on his breast. "Madame, I have the honour to address you. You see this man's wife; I ask you to perform operation on her. I will give you the things, I will tell you what to do. I have everything ready." I said, "I am a nurse; I do not operate." "Yes, madame, I know you not doctor; you surgeon." Thinking, perhaps, that it might be some simple thing that any obstetric nurse might do, perhaps mis-called an "operation," I asked for more particulars. I ascertained that the lady was a Purdah lady, and therefore she could not be allowed to see a male doctor. He explained that this poor lady was suffering from fibroid tumour of the uterus, and that it would be necessary shortly to perform Cæsarean section, and from what he had heard of me, he felt he could place every reliance in me. I scarcely grasped his meaning. At first it was too overwhelming. I then politely declined. He would take no refusal. He said, "Only danger, hæmorrhage." He would be near, I could ask him, he would tell me what to do—"just do what I tell you, all be well." I suggested the qualified lady doctor of the station. "No, madame, this Baboo and I have confidence in you, no confidence in her, she is a physician." My explanations and protestations were unavailable. He thought I was bargaining for fees; he then told me to name my own. "Baboo very rich man, money no object." I, then, finding that money was plentiful, suggested a neighbouring station about 150 miles off, where there were three lady doctors; and one of them, this year, very successfully performed Cæsarean section, and I knew that the Purdah lady could be attended too, if removed to that place, with all the privacy and delicacy necessary for a Purdah woman's case. They discussed the idea, and though disappointed with my refusal, agreed to consider it. Dr. Ram, before leaving, requested that I would at least go and see the patient. I agreed to do so and took down the address. Always ripe for adventure or an interesting case, I hired a gharry, as safer than my own carriage, and with a lady friend set off, longing to see the interior of the lady's house and the domestic arrangements of a wealthy middle class native's domain. We rattled away through cantonments, then through the civil lines, on past tumble-down huts into open country, with the fields of castor oil plants, waving Indian corn, fenced in with prickly pears and mud banks; here and there we had a peep down avenues of stately Oriental trees, and the tall palms stood out against the lurid red of the evening sky. We met a number of gharries and barouches belonging to native gentlemen and Baboos who were going for their evening drive in the park. Next we passed by a rajah's garden, with its women's quarters, temples, and palace. Then over the railway line and between paddy fields, till we came to a place where a fair was being held; how I longed to paint those vivid costumes and that motley crowd with the Eastern see-saws, a bogeyman about 30 feet high, merry-go-rounds, and what not. Then on we went past another rajah's garden and into the city at last. What a maze of picturesque and narrow streets, shops, houses, and temples in heterogeneous confusion, but nowhere could we find either Dr. Ram or our Purdah lady. We were sent to no end of doctors, all natives; every one said they knew Dr. Ram, but no one could tell us where he lived. We were driven out of the city in one direction out to the banks of one of India's sacred rivers, and there the stillness and grandeur of the scene consoled us a little for our disappointment. Here the pious Hindoo burns his dead and consigns them to the river and the crocodiles or alligators. So back again into the city we were obliged to go, through narrow streets, ill-lit with lamps, for darkness had set in, and after being three hours away on a fruitless expedition we reached home tired out. I wrote to Dr. Ram and apologised for not having fulfilled my promise. I have received a letter from him in which he says he will call on me in a few days and give me much information. I shall hope then to send you something interesting and scientific.

I am sorry to say my horse has been ill with a deep-seated abscess reaching down to the pleura. I sent it to the infirmary, and I went to pay it a visit. The Horse Infirmary is built in five rows of arched stalls, open to the air; supported by pillars in the second and third rows, like a succession of corridors, are the stalls. I strolled down the centre path, and on each side were the patients. Each had a placard fastened by it at the side of the stall, such as "F B. 150 R.A.; disease, lymphadenitis; diet, usual; result, ———." There were seven cases of abscess, one sprained tendon, one general debility, one lymphadenitis, and one with a disease that caused enlargement of the bones above the hoof—he was being fired. The horses ranged from a huge charger and the battery horses down to a little pony ten hands high. The diets I noticed were "bran," "laxative," and "usual." I then saw the dispensary, or surgery, where there was a fine case of instruments, and rows and shelves filled with bottles and drugs. Then I went into the office, when the veterinary lieutenant gave me his opinion of my horse. The horses are each seen to by their own syces, or grooms, and the whole are looked after by the farrier-sergeant of the battery, who is practically house-surgeon. It was a new idea to me seeing my equine friends in hospital, and hearing about it may interest others.

Two Indian nursing sisters are detailed for duty in the Isazai Expedition.

### Everybody's Opinion.

*[Correspondence on all subjects is invited, but we cannot in any way be responsible for the opinions expressed by our correspondents. No communications can be entertained if the name and address of the correspondent is not given, or unless one side of the paper only be written on.]*

### HOME FOR AN INCURABLE.

Mrs. E. Cropper, Tolson Hall, Kendal, writes as follows: Some of your readers may notice among your advertisements an appeal for a home for a woman with spinal disease. I should like to say that though a terrible sufferer she is an example of Christian patience and wonderfully cheerful. Her husband, who works in a boot shop, is worn out with nursing her as well as earning their living. Her legs have broken as she was moved in bed within the last three years. She has been 18 years bedridden. Her legs are in splints, and are rebandaged weekly. The doctor can do nothing for her, but injects morphia daily. He thinks she may live two years, perhaps more. The incurable hospitals we have applied to will not receive her because she is blind, and we have no trained nurse at our workhouse.

### UNIFORMS AS ADVERTISEMENTS.

"A Lancashire Matron" writes: I write to ask is there any remedy to prevent women who are not nurses wearing the uniform of nurses? For some time four girls have been going about this town dressed as nurses as an advertisement for some enterprising party. Each girl carries a bag with pamphlets, which she distributes to the passers by. On one bag is painted in white letters, "Digestive Food for Invalids and Infants." To my mind they might be dealt with for acting under false pretences. They are accompanied by a man. If the uniform is not to deceive, what is it used for? What does the nursing world think about it?

"ASSISTANTS' MANNERS AND NURSES' FEES."

A "Reader of The Hospital" writes:—What does Dr. Rainsford consider a *trained* nurse? A machine to carry out the doctor's orders without any reasoning in the matter, or one who, the more she knows and understands the reason for the treatment of his patients by the doctor, carries out his orders with intelligence, and, because of her superior knowledge and training, knows exactly where her duty ends and the doctor's treatment comes in, which she has conscientiously to carry out in his absence, having received his orders? Surely it cannot be the attempt at raising the standard of nursing in Dublin which causes the "nurses" of whom Dr. Rainsford has had experience to "be much too ready to interfere in the treatment of cases," it must be due to their own individual ignorance and bad training. I have always found that the higher up in his profession and the more intelligent a physician or surgeon may be, the more he expects the nurse to know and understand, and the more he depends on her reports of his case, and is not afraid of her having too much knowledge and therefore criticising his treatment. A good, well-trained nurse is always loyal to the doctor for whom she works, and unless there be this mutual confidence, it were better for the nurse not to undertake the case at all. Surely if we nurses want to read more about the treatment of cases we have only to buy books on the subject: short notes on the treatment in other hospitals, though very interesting and helpful, will not do much towards it. "In Dublin the best of trained nurses never ask for more than one guinea per week." Why is this? Surely if an educated woman has spent years in gaining experience and learning to be a thoroughly good nurse, her intelligent services are worth more than this! But does Dublin ask for such experience and knowledge in private nurses, or are those who engage them willing for their own good to pay for it? A very skilful Sister of the "Red Cross" Association is given as an example of the best of trained nurses in Dublin. I should like to know what is the term of training before admission to this association as a Sister. Is it two or three years at a good General Hopital, or, as is often the case, one year or even less is counted sufficient training for the responsible work of private nursing? Do the nurses take their own earnings—as Dr. Rainsford says this Sister attended his father for one guinea per week—or are they a clear profit to the association, receiving only a paltry salary themselves? I should think that the difference in salaries between London and Dublin is owing to the fact that a higher standard of nursing is demanded, and more value set on training in the former than the latter place. If more than one guinea per week is never asked, what society or hospital will be first to fix a higher standard for private nursing, and then demand higher salaries for their nurses, allowing them a fair commission on their earnings, and not taking all the profits for the association to which they belong?

### Appointments.

Withington Hospital, Manchester.—Miss Dora Robinson, charge nurse at the Hospital, Fir Vale, Sheffield, has been appointed Night Superintendent at the Withington Hospital.

Eastern Hospital, Homerton.—Mrs. Maud Evans has been appointed Night Superintendent at the above hospital. Mrs. Evans trained at Guy's Hospital, and afterwards went as Sister and district nurse in Dublin for nearly three years. Her next post was as district nurse in London for one year; since then she has been a charge nurse in the Eastern Hospital.

REAPING.

"As a man sows, so shall he reap," is a fact we have seen for ourselves in the fields, or heard of all over England lately. For the Harvest Homes and Harvest Festivals recall to our minds all the processes which have been gone through to bring us food to keep us alive. First, there was sowing the seed, and if that were good wheat, or barley, or oats, there was almost sure to be an excellent crop, whereas if weeds sowed themselves the farmer gained little but weeds in return. Then the land had been repaired, and afterwards kept in order while the blades were growing, so that with genial showers and bright sunshine the crops came to perfection, because there was a blessing sent from Heaven upon them. It is God which gives the increase. I wonder how many of us have thought that it is similar with our bodies and minds. We start in life with healthy bodies, at least most of us do, and pure minds, and it should be our duty and pleasure to keep them clean and healthy. But we are often so foolish, even reckless in youth, that we sow the seeds of all sorts of complaints of which we shall unfortunately reap the harvest one day, in consumption or some other fell diseae. Later in life the father or mother break down from overwork, or undue anxiety to supply comforts or perhaps daily bread for their children. When the mischief is done, we bewail ourselves and wish we had taken to heart the warnings which are always given us, for God working through nature tells us when we are taking liberties with ourselves. Why will not those who wear thin clothing and have bad colds and rheumatic pains take the hint? Cannot the darling pipe be used with discretion and not allowed to become a nuisance to others and a blight to ourselves? Other practices there are, which we will not name, but which, alas, bring ruin on body and soul.

Ah! dear friends, it would be cold comfort to tell us in our trouble that "as we have made our bed so we must lie on't." But we have something better to hear. There is a great and kind Father Who is very pitiful to His children, Who watches over them, draws them back from destruction, and sees, and encourages their faintest wish to do better. Let us use the time we are obliged to spend in idleness in making good resolutions for the future, not only for our bodies but for our minds also—as we can sow anger, impatience, wilfulness, broadcast, and a miserable harvest we reap, whereas if we try to be gentle, peaceable, patient, loving to those about us, we shall gain a rich crop of happiness. The Almighty does indeed give the increase to such farming as this. He sends the dew of the Holy Spirit, and the small rain of His blessings upon those who strive to serve Him. He makes His face to shine upon us, and we ripen under His life-giving beams, till we bring forth fruit, some sevenfold, some ten fold, some a hundredfold, to His glory.

### Glasgow Nurses "Off Duty."

On Thursday, the 27th ult., the Lord Provost of Glasgow, Sir John Muir, entertained the medical staff, officials, and nurses of the various hospitals in the city at the municipal buildings. The occasion was unique. It was not Sir John and Lady Muir as private individuals inviting certain doctors and nurses to spend an evening with them, but the head of the municipality of the largest city in Scotland—the second city in the Empire if you take the natives word for it—doing honour in his official capacity to the workers in the medical charities of the district. Consuls and councillors came to the reception, municipal and other local dignitaries, accompanied by their wives and daughters rustling in brocade and glistening lavishly with diamonds, but it was not they who were the guests of the evening. While all were received courteously with that frank and friendly manner which gives such a feeling of genuiness to the hospitality of the West of Scotland, the warmest welcome was given to the women in the simple cotton gowns—the most plainly dressed guests that ever graced these corridors or halls.

The municipal buildings of Glasgow are Venetian in their richness. The Mansion House is dim and poor compared with them. There is a great staircase of marble—white marble steps, marble balusters of a creamy yellow, walls panelled with brown and white marble, glistening marble pillars supporting the moulded arches which stretch in endless arcades with such effects of colour and perspective as would delight the soul of Alma Tadema. The reception-rooms are a marvel of costly woods carved with the finest skill, and delicately tinted hangings of silken tapestry. Only the great ball-room is as yet cold and colourless, with great wall-panels waiting until the energetic, clever, ambitious "Glasgow School," which has won its place in the art-world by talent and originality, not wholly free from daring eccentricity, shall have produced a Paul Veronese to paint them in fresco—waiting at least until the plaster shall be dry enough to permit such decoration. But here masses of flowers and palms, and the *va-et-vient* of 800 guests, gave colour and movement enough.

Among the palm-leaves that brightened hall and corridor one saw the various uniforms. The nurses of the City Hospital wore dark blue stuff gowns, brightened by a red ribbon at throat and waist, while the probationers were dressed in a pretty hair-line stripe of lilac and white. The Royal Infirmary nurses wore blue and white and pink and white striped galatea, according to their rank in the hospital, and those from the Maternity Hospital the same colours in checks. The uniform of the Western Infirmary is less effective—grey and red stripes on a white ground. The Hillhead nurses had one of the prettiest uniforms—blue cambric, with bird's-eye dots of white; but it was rivalled by the dark blue ginghams of the members of the Glasgow Sick Poor Nursing Association, made with pretty puffed sleeves. Considering the greater amount of muscular freedom which the arm can possess in a puffed sleeve compared with a tight-fitting one, it is remarkable that it is not more generally adopted. Miss Wood, the matron of this association, was present, dressed in black—as, indeed, were most of the matrons—with the blue badge of the Jubilee Nurses on her arm. The Lenzie nurses wore blue also also, but their aprons, instead of crossing the shoulder, had the bib kept in place by a gilt chain round the neck. The dark grey tweed of the Eye Infirmary nurses had rather a gloomy effect among the brighter gowns, but there was only one really unbecoming uniform, that of the nurses from Gartnavel Asylum. They wore a dull grey alpaca that somehow suggested horse-hair, and very shapeless and unbecoming muslin caps with a band of black velvet. It was pleasant to note what comely, kindly faces were thus crowned, but why should asylum nurses, whose patients are, as a rule so nervously sensitive to colour and grace, be expected to wear such very unattractive toilets? Indeed, one could not help noting how bright and healthy the nurses looked, and among them were not a few so pretty that one could well imagine some of the gaily-dressed ladies who mixed with them wondering if such complexions were to be cultivated by the hard work and plain food of hospital life.

The majority of the nurses kept their uniforms strictly plain, though some indulged in a spray of roses or chrysanthemums at the throat. One, indeed, went so far as a bouquet with long streamers of white satin ribbon, while another carried a white feather fan. Chatelaines, of course, there were in abundance, with the usual array of forceps and scissors shining in a way to make silver feel dull, and often brightened with a "red-cross" pincushion at the waist. It was something of a marvel that there was no need to call for the surgical skill and materials at hand, for a malicious tin-tack deserting the carpet edge it belonged to, just in front of the Lord Provost, one nurse tripped on the carpet and nearly fell upon Sir John Muir's ermined breast. Her presentation to the Lord Provost resembled the effusive etiquette of the Irish Viceroy's court rather than our colder Scottish ways.

When all the guests had been received, Sir John and Lady Muir led the way to the great hall, where Dr. Macewen, the newly-appointed Professor of Surgery, delivered an address, in which he spoke of the evolution of nursing, which he hoped to see duly recognised as a profession. He spoke appreciatively of nurses as they are, but expressed the opinion that a nurse could never be too highly trained. Professor Story also spoke, and congratulated Sir John Muir on the fact that he had ended his official life as Lord Provost of Glasgow by this entertainment to those whose lives were devoted to the relief of suffering and the practical exposition of the gospel of goodwill towards men. In which congratulation The Hospital begs most heartily to join, and ventures also to express the hope that the bright example of courtesy and hospitality which Sir John Muir has set will be followed, not only by his successor at Glasgow, but by those who hold similar positions in all our towns—that at last the hospital and those who work in it will be recognised as an integral part of the city, worthy of all the dignity, honour, and courtesy of civic life.

### Notes and Queries.

### Queries.

(19) *Books Wanted.*—Can anyone tell me the publisher of "Aids to the Injured and Sick," by Dr. Wilmingham Gills? also I wish to know if Dr. Fletcher Little, of the West End Hospital for Nervous Diseases, has written any book on massage?—*Nurse F.*

(20) *Boracic Acid, Paraffin.*—*J. E. C.*

(21) *Agnes Earl.*—Can anyone oblige Miss Hill, Children's Home, Witney, Oxford, with present address of this lady?

(22) *Strophanthus.*—1. Will you kindly tell me in your next "Answers to Queries" what is strophanthus; 2. Also whether it is correct to write "spoonfuls" or "spoonsful"?—*Sister Mary.*

### Answers.

(19) *Books Wanted* (*Nurse F.*)—We do not know the publisher, but we will inquire for you on both your questions, perhaps one of our readers will help you.

(20) *Boracic Acid Paraffin.*—We never prescribe even for the simplest complaint. Your first query is entirely a matter of health, and is not the simple affair people are apt to consider it, and you should consult a medical man.

(22) *Strophanthus* (*Sister Mary*).—1. Strophanthus is a new remedy introduced by Professor Thomas Frazer, of Edinburgh. It is a powerful cardiac tonic. 2. We believe "spoonfuls" to be the correct way of using the plural.

## Figures and Tables

**Figure f1:**